# Can GPs working in secure environments in England re-license using the Royal College of General Practitioners revalidation proposals?

**DOI:** 10.1186/1471-2296-13-123

**Published:** 2012-12-20

**Authors:** Jane Coomber, Rodger Charlton, Jill E Thistlethwaite, Liz England

**Affiliations:** 1 Primary Care Clinical Sciences, University of Birmingham, Edgbaston, Birmingham, B15 2TT, UK; 2 Division of Primary Care, School of Community Health Sciences, The Medical School University of Nottingham, Nottingham, NG7 2RD, UK; 3 Director of the Centre for Medical Education Research and Scholarship, The University of Queensland School of Medicine, 288 Herston Rd, Herston, 4006, Qld, Australia; 4 Primary Care Clinical Sciences School of Health and Population Sciences, College of Medical and Dental Sciences University of Birmingham, Edgbaston, Birmingham, B15 2TT, UK

**Keywords:** Revalidation, Re-licensing, Medical continuing professional development, General practitioners, Family physicians, Sessional GPs, Salaried GPs, Secure environments, Prisons

## Abstract

**Background:**

Revalidation for UK doctors is expected to be introduced from late 2012. For general practitioners (GPs), this entails collecting supporting information to be submitted and assessed in a revalidation portfolio every five years. The aim of this study was to explore the feasibility of GPs working in secure environments to collect supporting information for the Royal College of General Practitioners’ (RCGP) proposed revalidation portfolio.

**Methods:**

We invited GPs working in secure environments in England to submit items of supporting information collected during the previous 12 months using criteria and standards required for the proposed RCGP revalidation portfolio and complete a GP issues log. Initial focus groups and initial and follow-up semi-structured face-to-face and telephone interviews were held to explore GPs’ views of this process. Quantitative and qualitative data were analysed using descriptive statistics and identifying themes respectively.

**Results:**

Of the 50 GPs who consented to participate in the study, 20 submitted a portfolio. Thirty-eight GPs participated in an initial interview, nine took part in a follow-up interview and 17 completed a GP issues log. GPs reported difficulty in collecting supporting information for valid patient feedback, full-cycle clinical audits and evidence for their extended practice role(s) as sessional practitioners in the high population turnover custodial environment. Peripatetic practitioners experienced more difficulty than their institution based counterparts collating this evidence.

**Conclusions:**

GPs working in secure environments may experience difficulties in collecting the newer types of supporting information for the proposed RCGP revalidation portfolio primarily due to their employment status within a non-medical environment and characteristics of the detainee population. Increased support from secure environment service commissioners and employers will be a prerequisite for these practitioners to enable them to re-license using the RCGP revalidation proposals.

## Background

In the United Kingdom medical practitioners were awarded professional status under the 1858 Medical Act. This act established the General Medical Council (GMC), which was given responsibility for regulating medical education and professional discipline through a practice register and fitness to practise panels. Over the subsequent 140 years the GMC maintained this regulatory role, providing the medical profession with a substantial degree of autonomy over its affairs. However, a series of publicised medical scandals in the 1990s, including the arrest in 1998 of the serial killer and GP Harold Shipman, prompted the government of the day to demand a review of medical regulation [1]. In response to this request, the GMC voted in 1999 to extend its regulatory function from breaches of professional conduct in qualified doctors to proving their competence through periodic review of practice (ie revalidation) and re-licensing. The GMC’s original proposal for medical revalidation comprising evidence of completed sets of annual peer review documentation in a five yearly cycle was rejected in 2006 by Dame Janet Smith, Chair of the Shipman Enquiry, as lacking the necessary standards and objectivity [2]. Following a demand by the government for a more robust revalidation process to support medical re-licensing [3], the GMC put forward revalidation proposals consisting of a generic professional assessment from *Good Medical Practice* (GMP) framework of 12 attributes from four domains of clinical and professional competence against which a doctor’s practice could be appraised and objectively assessed [4].

Under the revised revalidation proposals, the Royal Medical Colleges were charged, on behalf of the GMC, with the responsibility of proposing the standards and methods of revalidation for doctors who practised their clinical specialism for 50% or more of the doctor’s workload. The Royal College of General Practitioners (RCGP) proposed that the revalidation process for GPs, who comprise one-quarter of the National health Service (NHS) medical workforce (24.87%, n=35,767) in England [5], should build on current annual peer review or appraisal (Table 1) with GPs presenting supporting information of patient-centred clinical practice and areas of extended practice at peer review (Table 2). The GPs will be responsible for collecting their supporting information and reflecting on this evidence as well as information from other local sources (eg. clinical governance). The supporting information would then be discussed as part of the formative peer discussion with the GP appraiser (a GP trained to undertake peer reviews), who ensures the quantity of the GP’s supporting information was appropriate for that point in the revalidation cycle and demonstrated the professional values set out in *Good Medical Practice*[6]. Evidence from the five peer reviews would then be forwarded in a five-yearly cycle to a responsible officer (RO), usually the medical director of the doctor’s revalidating supporting organisation, to make a recommendation to the GMC for re-licensing [7].

**Table 1:** Current GP peer review/appraisal

• Annual peer review was introduced for GPs in 2002
• These practitioners are obliged to register with a primary care organisation’s performer’s list
• The overall responsibility of a primary care organisation is to ensure the health needs of its local population are met
• One function of a primary care organisation is to administer an annual peer review for qualified doctors on its performer’s list
• GPs comprise GP principals and sessional GPs. GP principals are community based and contracted by NHS primary care organisation commissioners to provide general medical services for a registered community population. Sessional GPs (salaried GPs and GP locums) are employed by GP principals or other health service providers to deliver primary care treatment to a given population and are sub-contracted for a number of sessions per week (one session = one-half day of clinical practice).
• GPs are encouraged to prepare evidence of good practice on an electronic toolkit and/or paper documents to discuss at their annual review
• A GP’s evidence can be structured under the seven headings of *Good Medical Practice*[6] (the profession’s code of practice) headings of good clinical care, maintaining medical practice, teaching and training, relationships with patients, working with colleagues, probity and health
• The appraisal documentation is supported by clinical governance information
• Peers are trained by the primary care organisation to act as GP appraisers to further develop.
• GPs are obliged to change their GP appraiser every two – three years.

**Table 2:** **Overview of the supporting information under four generic headings that the RCGP proposes a GP submits in a five-yearly revalidation portfolio (December 2010)**[7]

**Generic heading**	**Supporting information**
General information	Personal details
	Scope of practice including extended practice*
	Contextual details
	Participation in annual appraisal, Personal Development Plan (PDP) and review of PDP
	Statement of probity and health
Keeping up to date	50 learning credits per year and 250 credits overall (one learning credit = one hour of learning activity including planning and reflection)
Review of Practice‡	Ten significant event audits (SEAs) including any serious incidents
	One full-cycle clinical audit
Feedback on practice**	One colleague survey (50% clinical colleagues, 50% non-clinical colleagues)
	One patient survey
	Review of complaints
	Compliments

*Evidence of extended practice = past and present medical qualifications in area of practice additional to GP’s main role and sign off from an appropriate colleague who has knowledge of GP’s extended practice role.

‡ GPs required to discuss audits’ findings at primary care team meetings.

** The GP, appraiser and RO must not have any involvement in collation of the results of the colleague and patient survey.

The RCGP was keen to ensure that their revalidation proposals were fair, accessible and achievable for all GPs in whatever capacity they were employed in the UK. Typically, GPs are community based and deliver general medical services to a registered community population, so the RCGP was interested to explore the feasibility of their proposed model of revalidation for the atypical group of GPs who delivered general medical services in secure environments to a detainee population. The main secure environments in the UK comprise prisons, secure mental hospitals, police custody suites and immigration removal centres. The latter mentioned institutions have grown up over the past 20 years to provide temporary accommodation for people awaiting deportation from the UK [8]. The vast majority of detainees are held in prisons (the England and Welsh prison population was 84,883 in April 2011 [9]) with approximate populations of 3000 – 4000 detainees in both secure mental hospitals [10] and immigrations removal centres [11]. Ninety-five percent of the prison population are male [9]. For police custody suites, 21/43 police services in 2006 reported a total of 428,434 patient contacts [12]. Detainees are a transient population with 38% of women and 28% of men receiving prison sentences for three months or less [13] and individuals rarely remaining in police custody suites for more than 24 hours [14].

Historically the prison service, which predates the NHS, directly employed doctors to provide health care for its detainees. However, concerns over the over-medicalisation of this patient population, who overwhelmingly present with mental health issues, substance misuse, communicable diseases and physical chronic diseases [13] led to a series of joint Prison Service and Department of Health reports around the time of the millennium. These documents identified evidence of doctors with skill decay practising in professional isolation in an environment where primacy was given to custodial management [15,16]. The reports recommended that doctors treating detainees should possess a postgraduate certificate in general medicine and participate in continuing professional development (CPD) organised through regional NHS Workforce Development Confederations to maintain their knowledge and skills in primary health care management. A further recommendation was that prison doctors should be encouraged to undertake a weekly session in general practice to reduce professional isolation [16-18]. From 2003, NHS primary care organisations (PCOs) have had the responsibility of commissioning health care for prisoners within their geographical boundaries with the aim of giving prisoners access to the same quality and range of health care services as the general public receives from the NHS.

At the time of our study, the commissioning of health care services for the immigration removal centre and police custody suites were in the process of transferring from the Department of Justice to regional NHS commissioners. The NHS continues to commission services for secure mental hospitals. The detainee commissioners contracted entire health care services to NHS providers (including local GP community practices) and non-NHS health providers, who in turn sub-contracted GPs to work sessionally (one session = one half day of clinical practice) in one or more secure environments dependent on the size of the detainee population [19]. In addition to working in residential custodial institutions, some GPs work part-time as forensic physicians (previously known as police surgeons) in police custody suites on an ‘on call’ basis [12]. The Department of Justice commissioners contracted a higher percentage of non-NHS health care providers than the NHS commissioners [8,12,13,20]. Doctors employed in an NHS secure environment facilities are required to be GPs and registered on a NHS PCO’s Performer’s List. The authors were unaware of the national number of GPs working in this GP sub-speciality.

With regards to the proposed GP revalidation proposals, findings from two RCGP revalidation studies had suggested that sessional or sub-contracted GPs could experience difficulties collecting the newer types of supporting information – patient and colleague surveys, significant event audits and full-cycle clinical audits – if they were peripatetic practitioners and lacked engagement and support from their employers and service commissioners [21,22]. In addition, one of the studies identified that as a group of participants, GP principals considered that the most difficult item of supporting information to source would be a sign off from an appropriate colleague to demonstrate competence for medical work they undertake regularly for remuneration outside their GP main role (ie. extended practice) [21].

The aim of this study was to explore the feasibility of GPs working either predominantly in secure environments as a sessional GP or as GP principals in an extended practice role in secure environments to collect supporting information for the RCGP proposed revalidation portfolio.

## Methods

A qualitative research approach with reference to frequency data was utilised to meet the study objectives for this study population which lacked an up-to-date sampling frame.

### Participants

We purposively invited GPs who worked either predominantly (50% or more of their workload) in secure environments and were expected to collect 11 items of supporting information in order to revalidate against the GP revalidation framework and a sub-cohort of community based GPs who would be expected to produce supporting information for their extended practice role from the 130 prisons, secure mental hospitals (high, medium and low), 10 immigration removal centres and custody suites of the 43 police forces in England secure mental hospital to participate in our study. Participants were recruited through email cascades to members of two national GP secure environment support group networks and employees of non-NHS secure environment service providers, attendance of project researchers at secure environment GP regional meetings and word of mouth between September 2010 and July 2011.

### Data collection

Exploration of secure environment GPs’ views on the feasibility of collecting supporting information for the RCGP proposed portfolio

For this national study, GPs were invited to participate in either a focus group or an individual semi-structured interview (face-to-face or telephone) of up to 60 minutes duration to gain an in-depth knowledge of GPs’ views of the RCGP revalidation proposals. The face-to-face interviews were audio recorded with the participants’ consent. The topic guide was designed by the study researchers in line with the study objectives and focused on the GPs’ views on current appraisal arrangements and the facilitators and barriers of collecting the RCGP proposed items of supporting information.

Feasibility of GPs to collect the RCGP proposed supporting information in secure environments

In addition, these GPs were asked if they were willing to submit some items of supporting information that they had collated in secure environments over the past 12 months (i.e. during the four month study data collection period and over the previous eight months) as guided by the current RCGP revalidation proposals to the research team and tell us of their experience of this process through completion of a GP issues log and a follow-up interview. Although the revalidation proposals suggested that GP principals who have an extended practice role in a secure setting should demonstrate they were competent in that role through their PDP and learning credits log entries and a sign-off of competency from an appropriate person, we were interested to explore if it was feasible for them to collect other types of evidence in that role. As the study was attempting to gain a snapshot of the ability of these GPs to gather five years of the proposed supporting information in a data collection period of four months, the study participants were asked to prioritise the collection of the newer types of supporting information - patient and colleague surveys, significant event audits and full-cycle clinical audits. The study researchers suggested the participants used the then current GMC draft patient [23] and colleague questionnaire for their feedback surveys [24]. The GPs received oral and written guidance on how to collect their supporting information and had access by email and telephone to members of the research team for the duration of the study.

The quantitative GP issues log with free text responses was designed and piloted by the study researchers and aimed to identify the ease with which the GPs had been able to collate their individual items of supporting information over the past 12 months (1 = very easy to 4 = not at all easy) and collect GP demographics of main workplace, extended practice roles, year of first GMC full qualification, years of experience working in secure settings, country of primary medical qualification and gender. Participants who submitted supporting information were invited to take part in a follow-up semi-structured face-to-face or telephone interview to gain an in-depth view of their experience of the facilitators and barriers of collecting supporting information for the RCGP revalidation portfolio in secure environments.

### Data analysis and ethical considerations

Excel software was used to calculate the quantitative data frequencies. The focus group and individual interviews were transcribed verbatim and then read by two researchers to identify and categorise themes [25]. Telephone interview notes were typed up by the researcher as soon as possible after the interview. Ethical approval was obtained from the Warwick Medical School Biomedical Ethics Committee. The GPs read the study information sheet and all subsequent questions were answered by the researchers prior to the participants signing a study consent form. Participants were assured of their anonymity and confidentiality of their data.

## Results

Exploration of secure environment GPs’ views on the feasibility of collecting supporting information for the RCGP proposed portfolio

### Participants

Thirty-eight out of 50 GPs (76%) who agreed to take part in the study participated in an initial interview. Twenty-four attended one of five focus groups (n = 2, n = 9, n = 3, n = 8, n = 2), four took part in a face-to-face interview and 14 participated in a telephone interview. Four of the GPs who participated in a telephone interview were subsequently present at a focus group.

(i) *GPs working predominantly in secure environments*

The majority of the interviewees (71%, n = 27) worked predominantly in a secure environment; of these 24 GPs worked in prisons, two in police custody suites and one in a secure mental hospital in nine out of the 10 National Offender Management Service regions of England (Table 3). Twenty-one of the prison GPs worked between two and 10 sessions per week in one prison. Three of the prison GPs regularly worked in a cluster of prisons (2 substance misuse specialists and 1 GP locum). Twelve of these GPs held management positions within secure environment settings. Seven GPs undertook clinical practice exclusively in a custodial setting and the remaining GPs undertook one to two clinical sessions per week in a general practice. In addition, one GP worked in an immigration removal centre and one was a forensic physician in an extended practice role.

(ii) *GP principals working in an extended practice role in secure environments*

**Table 3:** Illustrates the numbers of GP participants who were either predominantly employed in a secure environment or in an extended practice role as a GP principal within each of the four types of secure settings (n = 38)

**Secure environment (SE) employment type**	**prison**	**Secure mental hospital**	**Police custody suite**	**Immigration removal centre**	**TOTAL**
**GPs predominantly employed in SE**	24	1	2	0	27
**GP principals**	7	2	1	1	11
**TOTAL**	31	3	3	1	38

We recruited 11 GP principals, the majority of whom worked in prisons (n = 7) between one to five sessions per week, with two GPs undertaking one to two weekly sessions in a secure mental hospital, one GP working occasionally in an immigration removal centres and one GP that was on call 36 hours per week as a forensic physician.

Both set of GPs, whether they worked predominantly in secure environments or non-secure environments, had been medically qualified for a median of 22 – 23 years and spent a median of 4 – 5 years working in a secure environment (Tables 4 and 5). Overall, the majority of GPs were male (32/38) (Table 6) and the country of primary medical education was the UK.

**Table 4:** Length of years since first GMC qualification by GP participant employment type (n = 32)

**Secure environment (SE) employment type**	**Less than 10 years**	**11 – 20 years**	**21 – 30 years**	**Over 30 years**	**TOTAL**
**GPs predominantly employed in SE**	3	8	8	4	23
**GP principals**	1	2	4	2	9
**TOTAL**	4	10	12	6	32

**Table 5:** Number of years GPs worked in secure environment by employment type (n = 24)

**Secure environment (SE) employment type**	**1 year or less**	**2 – 5 years**	**6 – 9 years**	**Over 10 years**	**TOTAL**
**GPs predominantly employed in SE**	4	6	3	2	15
**GP principals**	1	4	0	4	9
**TOTAL**	5	10	3	6	24

**Table 6:** Gender of GP participants (n = 38)

**Secure environment (SE) employment type**	**Female**	**Male**	**TOTAL**
**GPs predominantly employed in SE**	8	19	27
**GP principals**	0	11	11
**TOTAL**	8	30	38

### Findings

Three key themes that emerged from the initial GP interviews were:

•Difficulties in collecting the newer types of supporting information were anticipated

•Inability of non-secure environment GP appraisers and ROs to interpret evidence for revalidation was a concern

•Current lack of employer and commissioner CPD opportunities

### Difficulties in collecting newer types of supporting information were anticipated

The majority of GPs considered they would have difficulty in collecting the data required for a patient survey and a full-cycle clinical audit.

*Patient survey -* There were issues around the administration of the survey and the ability of the detainees to complete the questionnaires. First, within custodial environments GPs had to gain permission from the organisation’s governor to carry out a patient survey. Second, there was a lack of personnel to administrate the survey and these practitioners were aware that handing out and analysing the questionnaires themselves could positively bias the results.

"I’m not supposed to be giving them to patients anyway … to hand pick people for these questionnaires… would have plenty of scope for intercepting the [completed] negative ones and putting forward the good ones. (41)"

The GPs generally felt that the custodial population, with lower levels of cognitive skills and English literacy and higher rates of mental health issues than the general population, may experience problems in understanding the questions and thus produce unrepresentative patient population feedback.

"… because there’s the literacy rate… there’s the language rate… the ones that can’t speak English at all… so I’m worried that I’m losing all that group of patients… (10)"

"Some [secure mental hospital patients] might be having a bad day (35)."

Also a small proportion of clients in police custody suites can be under the influence of substance intoxication or have sustained mental or physical injuries and may not be able to complete a questionnaire then or later if they cannot recall the medical consultation.

"Most able to do it and others could be sent a sheet later but not remember being in custody. (48)"

*Full-cycle clinical audit -* In the prisons and immigration removal centres, electronic medical record systems from which to draw off the audit data were evolving and there was a high turnover of detainee population. The GPs reported that the prison electronic medical record systems did not possess an accurate picture of patient population as NHS and custodial electronic medical record systems were not linked and new detainee notes were absent until the GP had obtained the detainee’s consent to request their NHS medical records from their own GP.

"Yes, when they come in… you have to get consent from the patient to contact the GP and then wait awhile…. (13)"

And departed detainees may not have been removed from the system.

"… if we tried to search on people in the prison who had asthma, last time we did it we got 4,600 patients. We only have 1500 in the prison at any one time… (34)."

The participants highlighted the rapid turnover in some of these prisons, especially the remand and local prisons that militated against producing full-cycle audits.

"If you’re trying to audit, for example, people who’ve had a heart attack and were on aspirin…most of them have left… I was on last night and we had 42 new patients come in… that means 42 have gone out that morning, you can’t audit, cos the turnover is so vast. (34)"

Even if the patient’s notes were on an electronic system the records may be incomplete as GPs working in secure environments still handwrite patients’ prescriptions and notes and Code Reading (ie. a coded thesaurus of clinical terms by which clinicians record patient findings and procedures in health and social care IT systems) can be poor in these establishments.

"…people are not read coding things, it’s hard to get information and I’ve got about 1000 patients… and at the moment we don’t do any prescribing [electronically], so none of the medication, you can’t search by medication. (13)"

Forensic physicians, who typically attended their patients on a one-off basis and then passed their handwritten patients’ notes over to their employing organisation, argued it would be challenging to produce a full-cycle clinical audit.

"Not sure how I would do this as a forensic physician in custody. Don’t keep the records. (2)"

In addition, the participants questioned if they would be able to carry out the clinical audit within their working sessions.

"Well, I’m contracted to do two sessions a week, those two sessions are full… then you come out of your [morning] clinic and you have to go to your 12.00 pm meeting… (24)"

*Colleague feedback -* Forensic physicians felt it would be challenging to carry out a colleague survey as they are isolated practitioners working intermittently alongside a changing population of police personnel.

"You are lone workers… police personnel different each time. (2)"

*Significant event audit* - A concern expressed by one GP was the misinterpretation of a significant event audit, an event which the doctor can learn from, as a serious incident which is then taken up formally by the custodial organisation.

### Inability of non-secure environment GP appraisers and ROs to interpret evidence for revalidation was a concern

Due to a paucity of GP appraisers with experience of working in a secure environment, some GPs working in these settings currently participated in peer reviews with GP appraisers who lacked specific knowledge of their specialist practice. These GPs were concerned, therefore, that having collected their supporting information, the GP appraisers without forensic experience may not be able to interpret their specialist practice evidence for revalidation.

"When you are doing forensic work, you have to be appraised by somebody in that professional sphere who understands what your role is and be able to appraise you appropriately, so I don’t understand how they are going to use general GPs to appraise prison GPs [for revalidation]. (27)"

They particularly feared that non-secure environment GP appraisers would negatively interpret their patient survey feedback. They argued that patient feedback in custodial settings may be poorer than that of their community peers as detainees did not experience patient continuity and choice of GP as a number of GPs typically visited one prison in a duty rota system. Also, the GP’s role within a custodial environment was not neutral.

"No matter how much you explain that you work for the NHS, which we do, you are automatically regarded as part of the system that incarcerated them, and so there is always this bad taste and bad intentions and bad feelings before we’ve even met. (37)"

Forensic physicians were aware that their medical diagnosis could affect a person’s liberty and conversely, diagnosis of a medical condition by an immigration removal centre GP would invalidate a person’s extradition order.

In addition, GPs’ reluctance to meet patients’ treatment needs as opposed to their wants being met may affect patient feedback, particularly in relation to requests for analgesics.

"If you certainly give them a survey like this and ask ‘how was the doctor, polite or and so on?’ they would say ‘No, he was absolutely awful because I wanted my Tramadol and he didn’t give it to me’. (6)"

### Current lack of employer and commissioner support for CPD opportunities

There appeared to be a limited choice of training opportunities for the GPs to maintain their knowledge and skills in their secure environment and generalist practice. A few prison GPs commented that they would appreciate access to ethical and security based learning as well as general medicine CPD. However, funding did not appear to be available to some of these GPs for CPD and protected study time was a low priority. For general medicine training opportunities, a significant number of GPs who worked predominantly in secure environments were not invited to local GP principal meetings.

"I’ve received nothing at all from the PCT to invite me to any meetings. I am not on any communication feed… It is very isolated. (29)"

For those who did receive invitations, the combination of travel time and time spent clearing security could be longer than the length of the meeting. They felt that they would be better able to attend one day events.

"…for a lunch time meeting, which when you’re in a prison is useless, because by the time you get yourself out and to where it is and back its taken three hours for a one hour meeting. So you don’t go because you just can’t nip anywhere… so I tend to go to ones where it’s a day, where I just take off and go to a day. They’re are much easier for me to go to, because I can block and get a replacement and go. (27)"

Furthermore, some GPs employed full-time in a secure environment were denied the opportunity of keeping their general medical core skills up-to-date by employers who did not embed a weekly community GP session in their employment contract. As the RCGP stipulates that secure environment GPs must undertake a generalist medical weekly session in order to re-license, this employment contract omission can prevent GPs practising in secure environments.

"As a full time doctor who works within prisons I still do not have this [community general practice session] as part of my contract and currently this fundamental part of my skill set is being neglected. This will obviously ultimately cause problems (arguably unfairly) with my revalidation. I am aware of one doctor who works within prisons who has already been informed that he should expect a referral to the RO if he does not get some GP experience. (12)"

### Items of information submitted by the GPS

#### Participants

Twenty GPs (40%) submitted items of supporting information to the research team.

(i) *GPs working predominantly in secure environments*

Fifteen of the GPs (75%) worked predominantly in secure settings; 13 in prisons, one GP in a secure mental hospital and another as a forensic physician. Ten of the prison GPs worked between two and 10 sessions in one prison. Three of the prison GPs regularly worked in a cluster of prisons (2 substance misuse specialists and 1 GP locum). Eight of these GPs held management positions within secure environment settings. Four GPs undertook clinical practice exclusively in a custodial setting. One of the prison GPs was a part time forensic physician on-call 30 hours per week.

(ii) *GP principals working in an extended practice role in secure environments*

Of the five GP principals who submitted supporting information, three worked two to five sessions per week in one prison, one worked two sessions in a secure mental hospital and the remaining GP was a forensic physician on call 36 hours per week.

Both set of GPs, whether they worked predominantly in secure environments or non-secure environments, were predominantly male (65%, n = 13/20) and had received their primary medical qualification in the UK (80%, n = 16/20). The median length of years since first GMC qualification was 20 (range 6 – 37 years) with a median of 3 years experience of working in secure settings (ranging from under 1 year – 27 years). Nearly half of GPs had two or more extended practice roles (45%, n = 9).

### Items of supporting information submitted

**Figure 1:**
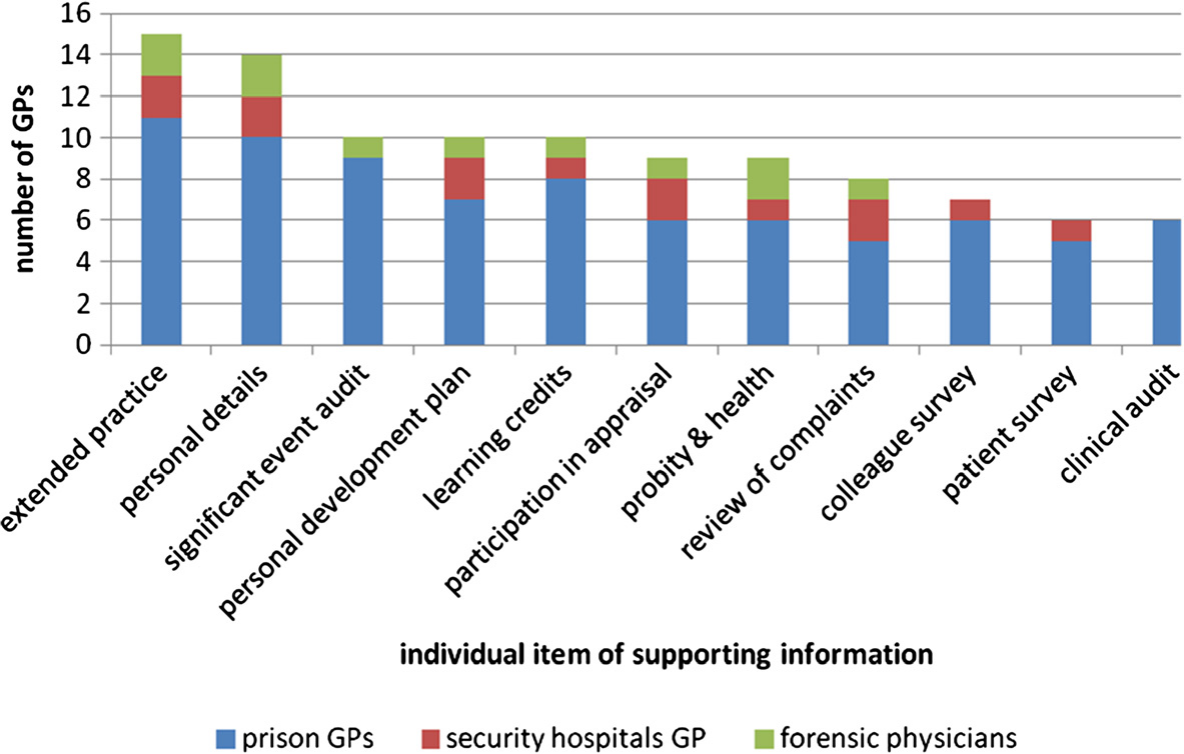
Individual items of supporting information submitted by GPs from three types of secure environments (n=17)

The GPs submitted between one and 11 items of supporting information to the research team. Overall, one-third (35%, n = 7) of the GPs produced evidence of colleague feedback and one-quarter (n = 6) produced a patient survey and a clinical audit (Figure 1).

(i) *Prison GPs*

The 10 GPs working predominantly in prisons collated supporting information for all 11 areas between them (the clinical audits were not full-cycle), managers and non-managers alike, and from those working as little as two weekly sessions in the custodial institution. Although nine participants submitted evidence of extended practice, only one participant presented a sign-off for competency in a substance misuse role with annual mental health peer review forms.

The three GP principals submitted evidence of extended practice, a colleague survey, a significant event audit and a clinical audit (not full-cycle). However, these practitioners did not submit a sign-off of their competency in their extended practice role.

(ii) *Secure mental hospitals*

The two secure mental hospital GPs submitted nine types of supporting information (not SEAs and clinical audits). The GPs working in a secure mental hospital submitted seven items of supporting information each regardless of whether they were predominantly employed in this secure setting or as a GP principal with extended practice role. The GP principal did not submit a sign-off of competency in their extended practice roles.

(iii) *Forensic physicians*

The two forensic physicians submitted eight items of supporting information (not clinical audits, patient and colleague feedback). The GPs working in this secure setting submitted five to six items of supporting information each regardless of whether they were predominantly employed in this secure setting or a GP principal. These practitioners did not submit a sign-off of their competency in their extended practice roles.

Ease with which GPs had collected items of supporting information over the past 12 months

**Figure 2:**
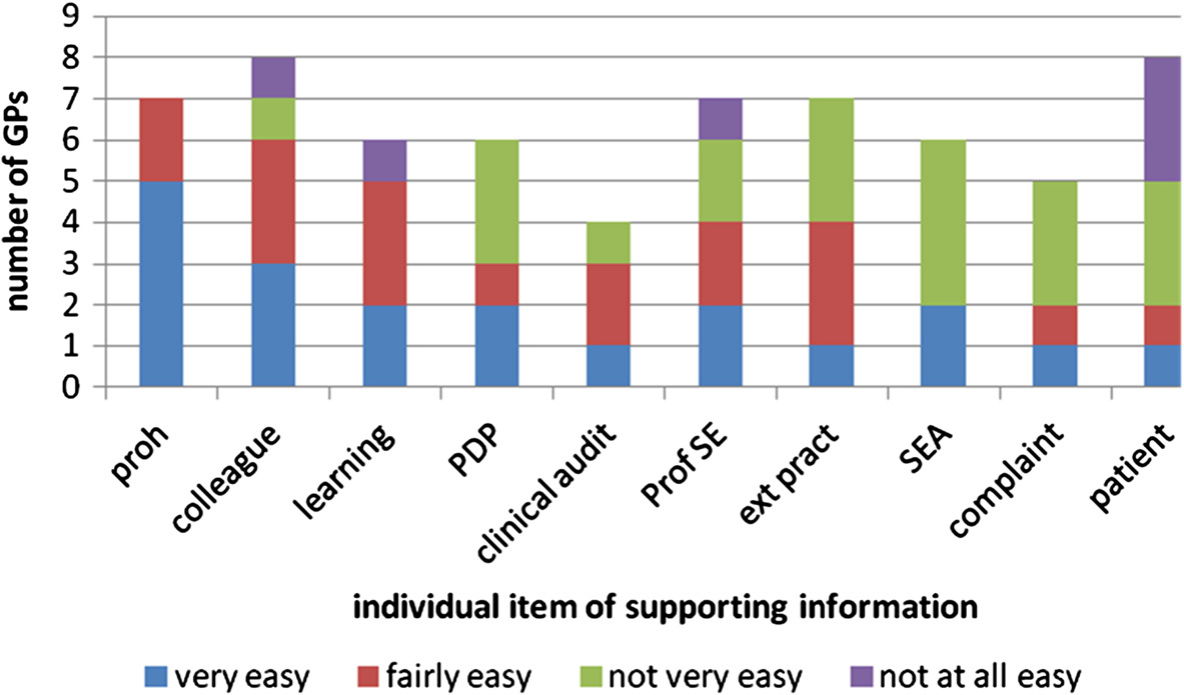
Ease with which GPs working in prisons collected items of supporting information over the past 12 months (n = 14)

**Figure 3:**
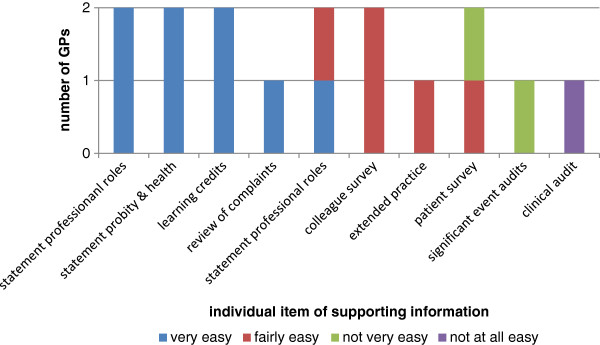
Ease with which GPs working in secure mental hospitals collected items of supporting information over the past 12 months (n = 2)

Seventeen of the GPs (85%) who submitted items of supporting information completed a GP issues log: 14 prison GPs, two secure mental hospital GPs and one forensic physician. The majority of prison GPs self-reported it was very easy or fairly easy to collect evidence for 7 out of the 10 types of supporting information and not very easy or not at all easy to collect evidence for SEAs, review of complaints and patient questionnaires (Figure 2). The two GPs working in secure mental hospitals reported it was very easy or fairly easy to collect evidence for 7 out of the 10 types of supporting information and not very easy or not at all easy to collect evidence for patient questionnaires, SEAs and clinical audit (Figure 3). Feedback provided by one forensic physician reported it was very easy to collect a statement of probity and health, learning credits, PDP and personal details.

### GP feedback on experience of submitting items of information

Nine GPs who had submitted a portfolio participated in a face-to-face interview (n = 4) or a telephone interview (n = 5). The remaining GPs forwarded their comments on a GP issues logs (n = 17) and/or via email to the study research team. Reasons given by the GPs for not submitting items of supporting information included the brevity of the data collection period, significant life events and the commercial and personal sensitivity of their supporting information.

Three key themes that emerged from the follow-up GP interviews and written feedback were:

•Barriers to collecting the newer types of GP supporting information

•Difficulty in producing a statement of competence for extended practice roles

•A time consuming process

### Barriers to collecting the newer types of GP supporting information

*Patient survey -* Five prison GPs successfully carried out a patient survey for our study. As the GPs had predicted, there was a lack of organisational support concerning the administration and analysis of the patient survey, except for one receptionist who handed out questionnaires to patients in one clinic. Interestingly, there was a positive bias in the GPs’ survey results.

"If I gave it [the questionnaire] out, more of them filled it in but they all said ’Oh yes, [the doctor’s] great’ and when the receptionist gave it out they were less, happier to say [the doctor] wasn’t great. (8)"

As expected, they found that a significant percentage of patients were unable to understand the questionnaire and might feel daunted by this task.

"Over a third needed help filling in the form because they can’t read or understand it… so they can either read a bit, seeing a form like that… it’s just scary… I think how you get round it, is that someone actually has to sit there and do it with them. (8)"

One participant did not consider it was feasible to carry out patient survey using an English questionnaire. In 40 consecutive consultations, this GP noted:

"Fifteen out of 40 prisoners in this foreign national prison would not have the English language skills to complete the survey. These 15 people spoke ten different languages. (5)"

In addition, these patient surveys had been all been carried out in routine clinics. The reception clinics (for in-coming institutional or police custody suite detainees) can be extremely hectic so there was no time to hand out questionnaires.

"Oh no, I wouldn’t do it for that [reception clinic], but this was just in our routine, routine GP clinics. (8)"

There was little time after the consultation to administer these questionnaires and it was preferable they were completed under the observation of healthcare staff.

"Not helped by prison staff who were keen to get inmates back to the wing and out of Healthcare, so did not allow them time to complete the forms. (24)"

"If they take the paper back to their cells, are they going to use that bit of paper for other things than writing on? I’d absolutely have to have agreement or not off the prisoner governors for that sort of thing. (7)"

Conversely to the GPs’ expectations, patient feedback was good except for a few cases where the GPs thought maybe the patients’ medical treatment and general wants has not been met.

"You see the two negative [feedback], I didn’t give them the medication that they wanted… (8)"

"Sometimes with a lot of ranting, complaints and items not related to my work but to the healthcare system in the prison. (6)"

One secure mental hospital doctor had received feedback on their practice derived from one question (s/he was the sole medical practitioner in that institution) of a 7-item general healthcare hospital questionnaire, which had been administered by the organisation’s personnel.

*Clinical audit* - The six prison GPs submitted a clinical audit, but they were not full-cycle and they appeared to have access to electronic medical records, possibly because they had focused on a relatively stable prison population. The GPs carried out their own audits with minimal assistance from custodial administrators.

"Yes, easy access to records because they're all computerized… I literally went into the records of 120 people…it was time consuming, to do a really valuable audit… (8)"

*Significant event audits and review of complaints* – There were established organisational systems of clinical governance in custodial environments. Some forensic healthcare teams had their established SEA-type meetings and others were moving towards this goal. For complaints, how they were processed varied between organisations.

"Complaints are looked at by the organisation, and put together thematically and fed back … (29)"

"… a complaint from either a police officer, from a patient or whoever makes the complaint, a solicitor, that something wasn’t done properly or the doctor had a poor attitude…. it gets categorized as a complaint….sent to the doctor .concerned to answer… (34)"

Generally speaking, as employed salaried GPs within a custodial institution, the study participants felt they were more of an invited guest within the custodial setting and did not have the same authority to ask fellow health care and custodial colleagues for assistance with collecting newer types of supporting information as they had as a GP principal. A working relationship needed to be established.

"I was a [GP principal] partner before I was in prison.. head of the pyramid. Because I was with my partners and we employed everybody else we met, and if I asked someone to do something it was their employer telling them to do it, so they would do it… I am an invited guest in a prison. I'm not in charge of any nurses, they've got their own gaffer who will tell them what to do, I, if I want anything done, have to rely on good favour and charm to get anything done. (27)"

### Difficulty in producing a statement of competence for extended practice roles

The GP principals and secure environment GPs were able to demonstrate they were up to date and fit to practise in their various extended practice roles through their PDP and learning credits log entries, but they experienced difficulty in identifying someone to sign off their competence in these roles. Some employment agencies produce negative and/or organisational GP feedback to their out-of-hours (OOH) GPs, but not individual doctor feedback.

"I am also a salaried GP in OOH services and as a quality requirement for that service they undertake patient satisfaction surveys and they do it monthly. I have been asking for many years to have this information collated in a doctor specific way so that it would assist appraisal. It still does not happen and this is a separate service. It seems to be a problem of salaried GPs working in orphan services generally. (12)"

As GPs are independent practitioners there was not always a clinician to provide a statement of competence. However, some GPs had taken the initiative and either undertaken two annual appraisals – one GP appraisal and one in their speciality field of practice (eg. one substance misuse practitioner participated in a mental health peer review) or undertaken a mini-appraisal with a peer within their forensic speciality.

### A time consuming process

The GPs felt that collecting the proposed revalidation supporting information was time consuming, beginning with understanding the type of information they were expected to produce and then sourcing and completing the appropriate templates; time they would rather spend with the patient.

"I find all this very time consuming, and I think this time should be counted as personal development time because I have to do this, and I am spending time on thinking how to count the credits, how to write reflections, how to record an impact (there are a few types of impact according to guidelines), what is significant event, what is whole audit cycle, how to find proper templates and how to fill in them and all this other things. This is so much paper work and I have to learn how to do this properly. I really would prefer to spend this time on clinical activities and clinical learning. (3)"

The GPs said they would appreciate organisational assistance with sourcing an appropriate ePortfolio, colleague and patient questionnaires and guidance with significant event analysis, clinical audits and feedback on complaints for revalidation.

Most of the GPs had collected their supporting information outside their clinical sessions.

"I have to do it really in my own time, I have to go in and do it either at a separate time from my clinic or stay after. (7)"

## Discussion

GPs working in secure environments reported difficulty in collecting supporting information for valid patient feedback, full-cycle clinical audits and evidence for their extended practice role(s) as sessional practitioners in the high population turnover custodial environment with peripatetic practitioners experiencing more difficulty than their institution based counterparts collating this evidence. GP principals reported difficulty in collating all the proposed supporting information for their extended practice role in secure environments.

Of the 11 items of supporting information that the RCGP proposes that GPs who work predominantly in secure environments collect for revalidation, our study GP participants reported they were most concerned about their ability to collate valid patient feedback and produce full-cycle clinical audits. Their fears concerning their ability to find a third person to administrate the patient questionnaires and that a significant minority of their patients could not understand the questionnaire were realised. As the GPs did not manage their health care team, they did not have the authority to ask a non-medical colleague to carry out this task [19]. Also, a significant proportion of the detainee population have learning difficulties (20% - 30%) and English may not be their first language (13% of detainees are foreign nationals in English and Welsh prisons [13]), and so may experience difficulty in understanding the patient questionnaire. A national or regional source of validated accessible patient questionnaires for detainees would be a useful resource for GPs working in secure environments. In addition, the GPs were concerned that incarcerated patients who believed their health and personal needs were not being met and lacked the choice of GP and continuity of care experienced by their community counterparts might give poor feedback. Their fears about poor feedback from detainee wants not being met may have been realised in a few cases, but the vast majority of the GPs’ patient feedback was very good.

Poor IT infrastructure in custodial settings and a high detainee population turnover appeared to be barriers to GPs producing full-cycle audits. The IT facilities varied between custodial institutions, with a few having no IT access. This study has highlighted the problems that GPs who work outside general practice, including those working in OOH, experience accessing patient medical records. The recent establishment of an internal prison electronic patient medical record system may be a precursor to the linking of NHS electronic patient record systems to these non-medical institutions. However, some of the GP participants were already carrying out quality improvement exercises such as small organisational audits and benchmarking practice against national professional guidelines (eg. NICE guidelines). Forensic physicians may have to carry out personal audits as they do not have access to patients’ medical records [26].

For significant event audits, the GPs were documenting significant events, but they were in the process of setting up meetings to discuss these events; once these meetings are established at the health care team level and the employers understand their purpose of GP self-development, collection of this item of supporting evidence may become easier.

The final item of supporting information that the prison GPs reported as not very easy to collect was review of complaints. As detainees are more litigious than their counterparts in the community the complaint policies are well established in secure settings [19]. However the complaints lodged can be non-clinical and non-GP specific, and as many of our GP prison participants were employed in large prisons, the complaints tended to be analysed at an organisational level before being forwarded to the relevant GP. For the GPs, where they were the sole health care practitioner, the GP complaint identification process was more direct.

One of the aims of a custodial institution is to improve health and social outcomes for adults through access to medical services for their detainees who, as a social group, do not traditionally access primary health care services in the community [27]. Therefore, secure environment employers should be supporting the GP revalidation process which is intended to ensure a high standard of medical treatment in their establishments.

The study’s forensic physicians, as practitioners working in reception-type clinics and lacking access to organisational patient medical records, either electronic or paper, did not submit patient or colleagues surveys or a clinical audit to the research team. In contrast to the GPs who work in the other two types of custodial workplaces, they were not part of a core on-site health care team with colleagues who could assist them to administrate patient questionnaires and complete colleague questionnaires or discuss SEAs and clinical audit findings. However, these participants were involved in educational groups, where they discussed clinical and professional issues. At the time of our study it was not clear if GPs who predominantly practised as forensic physicians would be revalidated against the GP specialist framework as GP appraisers can only sign-off competence in general medicine.

Moving on to sign-off of competence in an extended practice role, the GP principals were not easily able to identify an individual who could perform this task. A few GPs said that they use the same medical skills in secure environments as they did in general practice, but others disagreed and argued that they use specialist medical skills in this environment. There are clinical leads in secure environments that could carry out this task but it was not clear what type of evidence the GP should submit to prove competency. Was an appropriate colleague’s signature sufficient or, as the GMC now recommends that the doctor demonstrates evidence for their whole practice [28], should the GP produce several items of supporting information for their extended work role? Our study has suggested that GP principals may be able to collect several items of supporting information in an extended practice role in secure environments.

Continuing with the theme of extended practice role for GPs working predominately in secure environments, these doctors could have two or three extended practice roles. One of the issues raised by these portfolio practitioners is that a proportion of GPs worked almost equal amounts of time in their multiple work roles; in this scenario, as the RCGP proposes that the majority of supporting information the GPs collects will be in their ‘main role’, a fair chunk of their practice in extended practice roles will not be evaluated. However, multiplying the amount of proposed data to be collected would be extremely time consuming and onerous for these GPs.

As with the sessional community based GPs in previous revalidation RCGP studies, there was a suggestion that the communication link between sub-contracted GPs and the PCT was poorer than those experienced by GP principals who were directly commissioned to provide primary health care services, which was not off-set by the doctor’s employer. The lack of authority of these GPs in their work environment, whether in a medical or non-medical setting, does affect the feasibility of these GPs to produce the proposed revalidation supporting information. The RCGP recognises that secure environment GPs have non-standard careers and that they should be supported by their employers to gather their supporting information. They indicate that alternative methodology for accumulating supporting informational may be used if appropriate and acceptable to the doctor’s GP appraiser and RO [26]. As one of the aims of a custodial institution is to improve health and social outcomes for adults through access to medical services for their detainees who, as a social group, do not traditionally access primary health care services in the community [27], secure environment employers should be supporting the GP revalidation process which is intended to ensure a high standard of medical treatment in their establishments.

Although the aim of the study was to explore the feasibility of GPs working in secure environments to collect the proposed RCGP revalidation supporting information, the study highlighted the GPs’ discontent with current CPD arrangements in secure environments. The rhetoric of recent UK health care reports argued for health care professionals to be competent and up-to-date to deliver effective health care to its patients [14-16,29], but CPD continues to be patchy in secure environment settings. The paucity of local GP appraisers with secure environment experience may be due to the relatively small size of the UK custodial population, but PCTs could encourage more GPs working in secure environments to become GP appraisers. These dual practice GP appraisers could sign-off competency in the secure environment and general medicine. Inclusion of secure environment GPs in the planning and development of GP postgraduate education and delivery at a sub-regional level may result in increased access to these practitioners to local CPD events which would simultaneously reduce the professional isolation still keenly felt by these GPs. In addition, many of the study GPs reported that they had to prompt their employers to offer a weekly session in general practice to ensure they maintained their generalist skills. GPs who worked full-time and were not offered a general practice session in their employment contract often worked sessions on top of their day job to maintain their generalist skills. The commissioners should insert clauses in health care service providers’ contracts that stipulate evidence of CPD activities that promote both secure environment specific and general medicine competences and guarantee access to a weekly clinical general practice session to ensure these GPs are able to re-license.

The main strength of the study was the geographical and proportional representation of study GP participants from the four types of custodial settings in England in the initial GP interviews. The study also provided an up-to-date snapshot of GP occupational patterns and CPD opportunities in English secure environments. One limitation of the study was the short length of the data collection period in which to ask GPs to submit supporting information; unfortunately the recruitment phase of the study lasted longer than we anticipated. The novelty element of collecting supporting information that involved colleagues and the detainees and the unfamiliarity of performing these tasks using invalidated evidence collection tools and developing guidelines coupled with an absence of a system to support the collection of data may have discouraged some of the study GPs producing this supporting information for the study. Other limitations were the small size of the sub-groups that submitted items of supporting information and lack of evidence submitted by GPs working in immigration removal centres. Finally, as with all self-selected study participants, these GPs were very motivated individuals and may not be representative of their peers.

## Conclusions

GPs working predominantly in secure environments were able to collect general information for their main work role but experienced difficulty with collecting valid patient feedback and full-cycle clinical audits, which they argued was primarily due to their lack of management status within the health care team, primacy of security over detainee health care needs in a custodial environment and a high turnover detainee population. GPs who worked as core members of a health care team in prisons and secure mental hospitals with relatively stable populations reported it was easier to collect their supporting information than forensic physicians who were sole clinical practitioners working with temporarily detained persons in police custody suites.

GP principals experienced difficulty with submission of sign off for their competency in an extended practice role. There was uncertainty around who could carry out this task and the amount of evidence that the GP should provide as proof of competency in this role for revalidation. For the GPs working predominantly in secure environments, they tended to have more than one work role in secure environments as detainee populations were smaller than community registered population, and therefore did not fit with the RCGP revalidation proposed model of the main role and extended practice divide of supporting information.

In addition, the study highlighted that recommendations from national prison reports written ten to thirteen years ago aimed at improving the standard of detainee health care in secure environments have only been partially implemented. GPs now deliver medical treatment in these institutions but their specialist and general medical CPD have a low priority and some GPs do not have access to a weekly community general medicine sessions. Under the current RCGP revalidation proposals, GPs in custodial settings will experience difficulty re-licensing unless their employers and employees in secure environments support them in this professional development exercise though facilitating the patient feedback survey process, continuing to improve the electronic patient medical records system, provide access to secure environment specific and general medicine CPD, give a higher priority to protected study time and allocate time for a weekly community general practice session within the GP’s contract. The PCTs or their successor, the National Commissioning Board from April 2013, could insert clauses into the service providers’ commissioning contracts to ensure the health service providers promote an environment as outlined above to support the revalidation process for GPs.

## Competing interests

The authors declare that they have no competing interest.

## Authors’ contributions

JC, RC, JET and EE participated in the design of the study. JC was responsible for the data collection and JC and JET analysed the data. JC wrote the first draft of the article and incorporated the comments from RC, JET and EE who reviewed the draft article twice. All the authors have read and approved the final manuscript. RC was the project Principal Investigator and JC the project Research Assistant.

## Pre-publication history

The pre-publication history for this paper can be accessed here:

http://www.biomedcentral.com/1471-2296/13/123/prepub
